# Junctophilin-4 facilitates inflammatory signalling at plasma membrane-endoplasmic reticulum junctions in sensory neurons

**DOI:** 10.1113/JP281331

**Published:** 2021-03-03

**Authors:** Alexandra Hogea, Shihab Shah, Frederick Jones, Chase M. Carver, Han Hao, Ce Liang, Dongyang Huang, Xiaona Du, Nikita Gamper

**Affiliations:** 1 School of Biomedical Sciences, Faculty of Biological Sciences, University of Leeds, Leeds, UK; 2 Department of Cellular and Integrative Physiology, University of Texas Health San Antonio, San Antonio, TX, USA; 3 Department of Pharmacology, Hebei Medical University, Shijiazhuang, China

**Keywords:** bradykinin, dorsal root ganglion neuron, junctophilins, orai1, stromal interaction molecule 1

## Abstract

Junctions of endoplasmic reticulum and plasma membrane (ER-PM junctions) form signalling nanodomains in eukaryotic cells. ER-PM junctions are present in peripheral sensory neurons and are important for the fidelity of G protein coupled receptor (GPCR) signalling. Yet little is known about the assembly, maintenance and physiological role of these junctions in somatosensory transduction. Using fluorescence imaging, proximity ligation, super-resolution microscopy, *in vitro* and *in vivo* gene knockdown we demonstrate that a member of the junctophilin protein family, junctophilin-4 (JPH4), is necessary for the formation of store operated Ca^2+^ entry (SOCE) complex at the ER-PM junctions in rat somatosensory neurons. Thus we show that JPH4 localises to the ER-PM junctional areas and co-clusters with SOCE proteins STIM1 and Orai1 upon ER Ca^2+^ store depletion. Knockdown of JPH4 impairs SOCE and ER Ca^2+^ store refill in sensory neurons. Furthermore, we demonstrate a key role of the JPH4 and junctional nanodomain Ca^2+^ signalling in the pain-like response induced by the inflammatory mediator bradykinin. Indeed, an *in vivo* knockdown of JPH4 in the dorsal root ganglion (DRG) sensory neurons significantly shortened the duration of nocifensive behaviour induced by hindpaw injection of bradykinin in rats. Since the ER supplies Ca^2+^ for the excitatory action of multiple inflammatory mediators, we suggest that junctional nanodomain Ca^2+^ signalling maintained by JPH4 is an important contributor to the inflammatory pain mechanisms.

## Introduction

Calcium signalling in mammalian cells is very complex. Cells possess a multitude of Ca^2+^ entry and release pathways and express numerous Ca^2+^-sensitive proteins, which have distinct, sometimes even opposing, functional roles, and thus there is a necessity for Ca^2+^ signalling to be spatiotemporally restricted. Such a requirement is particularly evident in somatosensory neurons. Peripheral stimulation excites these neurons locally by activating their Ca^2+^ permeable sensory ion channels or G protein coupled receptors (GPCR) linked to Ca^2+^ signalling pathways. These neurons then generate and transmit action potentials linked to well-defined sensations (touch, temperature, pain, etc.). Yet, each neuron expresses numerous Ca^2+^ channels; hence, the intracellular Ca^2+^ signal fidelity is key to faithful somatosensory signal transduction.

Ca^2+^ signalling is indeed often restricted to local sub-cellular areas called micro- or nanodomains (Berridge, 2006; Parekh, 2008). Junctions between the plasma membrane (PM) and the adjacent endoplasmic reticulum (ER) are increasingly recognised as such Ca^2+^ signalling nanodomains (Berridge, 2006; Stefan *et al*. 2013; Jin *et al*. 2016; Pacheco *et al*. 2016). PM components of these ER-PM junctions often include GPCRs that induce Ca^2+^ release from the ER via activation of phospholipase C (PLC), generation of inositol 1,4,5-trisphosphate (IP_3_) and subsequent activation of the IP_3_ receptors (IP_3_R) localised at the ER side of the junction (Jin *et al*. 2013, 2016; Stefan *et al*. 2013). ER-PM junctions also harbour store-operated Ca^2+^ entry (SOCE) complex, which, in concert with the ER-localised sarcoendoplasmic reticulum Ca^2+^ ATPase (SERCA) is responsible for the maintenance and refill of the ER Ca^2+^ stores (Qiu & Lewis, 2019; Taylor & Machaca, 2019). In mammalian cells the main components of the SOCE complex are the STIM1-2 proteins (ER Ca^2+^ sensors) and the Orai1-3 proteins (pore-forming sub-units of the Ca^2+^ release activated Ca^2+^ channel, CRAC; Qiu & Lewis, 2019). ER Ca^2+^ release induces STIM1 oligomerisation at ER-PM junctions, where it binds to and opens PM-resident Orai1 channels (Qiu & Lewis, 2019; Taylor & Machaca, 2019).

In pain-sensing (nociceptive) sensory neurons, junctional nanodomain signalling is involved in generation of inflammatory pain by inflammatory mediators such as bradykinin (BK), proteases, histamine, prostaglandins etc., which are capable of exciting nociceptors via their PLC-coupled GPCR (reviewed in Linley *et al*. 2010; Petho & Reeh, 2012; Jin *et al*. 2016). BK is a ‘prototypic’ inflammatory mediator with robust neuronal and vascular action; it is produced at the site of tissue damage/inflammation by the kinin–kallikrein system (Petho & Reeh, 2012). Acting via B_2_ receptors (B_2_R), BK excites peripheral nerves via sensitisation of thermo-sensitive TRPV1 channels, inhibition of anti-excitatory M-type K^+^ channels and activation of Ca^2+^-activated Cl^–^ channels (CaCCs) (Brown & Passmore, 2010; Petho & Reeh, 2012, 2012; Jin *et al*. 2016). The latter two excitatory mechanisms are mediated, at least in part, by the ER Ca^2+^ release (Liu *et al*. 2010; Shah *et al*. 2020). Functional SOCE complex in dorsal root-ganglion (DRG) sensory neurons have been reported (Gemes *et al*. 2011; Munoz & Hu, 2016). Thus, CRAC channels are fully functional in DRG neurons and *I*_CRAC_ is enhanced after nerve injury (Gemes *et al*. 2011). STIM1 and 2 and Orai1 and 3, but not Orai2, were identified in DRG neurons and, interestingly, Orai1 and 3 activation was shown to increase excitability in these cells (Wei *et al*. 2017).

It is still not clear how the ER-PM junctions are formed, maintained and regulated but some scaffolding proteins localised between the ER and PM membranes were identified, including junctin, mitsugumin, sarcalumenin and junctophilins (Hadad *et al*. 1999; Srikanth *et al*. 2012; Takeshima *et al*. 2015; Correll *et al*. 2017). Here, we hypothesised that SOCE is necessary for maintenance of inflammatory GPCR signalling in sensory neurons. We further hypothesised that a structural maintenance of the ER-PM junctions is necessary for functional SOCE complex formation. While testing these hypotheses we discovered that in DRG neurons junctophilin-4 (JPH4) is necessary for ER-PM junctional microdomain integrity, ER Ca^2+^ store refill and for the maintenance of bradykinin-induced pain *in vivo*.

## Methods

### Ethical approval

All animal work carried out at the University of Leeds was approved by the University of Leeds Animal Welfare Ethical Review Body (AWERB) and performed under UK Home Office License P40AD29D7 and in accordance with the regulations of the UK Animals (Scientific Procedures) Act 1986. Animal experiments performed in Hebei Medical University were in accordance with the Animal Care and Ethical Committee of Hebei Medical University (approval number: IACUC-Hebmu-2020007). All animal experiments were performed in accord with the International Association for the Study of Pain (IASP) guidelines and conform to the principles and regulations as described in *The Journal of Physiology* (Grundy, 2015).

### Cell culture, transfection and siRNA delivery *in vitro*

Rat DRG neurons were dissociated and cultured as described previously (Liu *et al*. 2010; Jin *et al*. 2013; Kirton *et al*. 2013). In brief, male Wistar rats (7–14 days old; bred locally at the University of Leeds animal facility) were killed by isoflurane overdose, followed by cervical dislocation. Rats were decapitated, the spine removed and sectioned longitudinally. DRG were removed and dissociated in HBSS containing 10 mg/ml dispase (Thermo Fisher Scientific, Loughborough, UK) and 1 mg/ml type 1A collagenase (Sigma-Aldrich, Gillingham, UK) in a humidified incubator at 37°C and 5% CO_2_ for 13 min. Ganglia were then gently triturated and re-incubated for a further 2 min, followed by a final trituration and addition of ice-cold Dulbecco’s modified Eagle’s medium (DMEM-GlutaMAX, Life Technologies, Warrington, UK) supplemented with 10% fetal calf serum (FCS), penicillin 50 U/ml and streptomycin 50 *μ*g/ml (all from Sigma-Aldrich). Cells were then washed twice by centrifugation, resuspended in pre-warmed media and plated on 10 mm glass coverslips pre-coated with poly-d-lysine (Millipore Ltd, Watford, UK) and laminin (Insight Biotechnology, Wembley, UK). Cells were then incubated for 4 h to facilitate adhesion; each coverslip was then supplemented with 1 ml culture media and incubated for a minimum of 48 h prior to experiments.

For gene silencing experiments, small interfering RNA (siRNA) oligos against JPH4 were transfected into the DRG with Lipofectamine RNAiMAX Transfection Reagent (Thermo Fisher Scientific). DRG neurons were dissociated as previously described; 48 h post-seeding (approx. 70% confluence) the cultures were washed with fresh pre-warmed DMEM (without serum and antibiotics). The transfection reagent and siRNA were mixed in OptiMEM I reduced serum medium (Life Technologies) as per manufacturer’s instructions. The diluted siRNA-lipid complex was then added to cells followed by incubation at 37°C for 1 to 3 days. Two different siRNA products against JPH4 (Silencer Select, s175513 and s175511, Life Technologies) as well as scrambled control (Silencer Select Negative Control No. 1, Life Technologies) were used at 10 pmol per well in both single use or in combination.

Human embryonic kidney 293 (HEK 293) cells were cultured on 100 mm culture dishes in DMEM-GlutaMAX supplemented with 10% FCS, 50 U/ml penicillin and 50 *μ*g/ml streptomycin and kept in a humidified incubator (set at 37°C and 5% CO_2_). Cells were passaged every 3 days. HEK293 cells were transfected with flag-tagged JPH constructs in the 24 well plates using Fugene HD (Promega, Southampton, UK) according to the manufacturer’s instructions. The rat JPH cDNA constructs subcloned into pcDNA3.1+/C-(K)DYK plasmid were purchased from Genscript (Leiden, the Netherlands); JPH1 (NM_001106630.1, Genescript product code ORa01827D), JPH2 (NM_001037974.1, Genescript product code ORa09562D), JPH3 (NM_001107437.1, Genescript product code ORa03492D), JPH4 (NM_001003711.1, Genescript product code ORa09887D). Immunohistochemistry experiments were performed 48 h post-transfection.

### Fluorescence Ca^2+^ imaging

Plated DRG cultures were loaded with fura-2 by 1-h incubation with a mixture of 2 *μ*m fura-2AM (Thermo Fisher Scientific) and 0.01% pluronic acid (Sigma-Aldrich) in extracellular (EC) bath solution of the following composition (in mm): 160 NaCl, 2.5 KCl, 1 MgCl_2_, 2CaCl_2_, 10 HEPES, 10 glucose (pH 7.4). The mixture was then removed and cells were washed with EC solution. Fragments of coverslips were placed on the microscope perfusion chamber and EC solution was perfused over the cells through a gravity driven perfusion system at an approximate rate of 2 ml/min. The compounds were diluted in EC solution to the required concentration and applied via the perfusion system. The Ca^2+^-free EC contained (in mm): 160 NaCl, 2.5 KCl, 1 MgCl_2_, 1 EGTA, 10 HEPES, 10 glucose (pH 7.4). Fluorescence imaging was performed on an inverted Nikon TE-2000 microscope connected to a Till Photonics fluorescent imaging system (Thermo Fisher) comprising a Polychrome V monochromator and an IMAGO CCD camera. TillVision 4.5.56 or Live Acquisition 2.2.0 (FEI, Thermo Fisher) were used for image acquisition and processing. Cells were excited at 340 nm and 380 nm (50 ms exposure) and the emission was collected using a UV-2A filter (Nikon UK Ltd, Surbiton, UK). Regions of interest (ROIs) were used to select neurons from snapshots taken to record from and for further *post hoc* analysis. Recordings made were analysed in Microsoft Excel and Origin software.

### Immunohistochemistry

DRG were collected, washed with PBS and fixed with 4% ice-cold paraformaldehyde (PFA, Sigma-Aldrich) for 1 h. The ganglia were then embedded in 10% gelatine (Sigma-Aldrich) solution prepared with distilled water. The gelatine was then cut into 1 cm^3^ cubes containing a single ganglion, and further incubated in 4% PFA for 6–10 h at 4°C. The cubes were washed with PBS, cut to 40 *μ*m sections using a microtome (Leica VT 1200S, Leica Biosystems, Milton Keynes, UK) and washed three times with PBS. Sections were then incubated with a blocking buffer (containing 0.05% Tween 20, 0.25% Triton X-100 and 5% donkey and/or goat serum in PBS; all products from Sigma-Aldrich) for 1 h at room temperature. Subsequent incubation was done overnight at 4°C with a solution containing one or two primary antibodies (Table 1), depending on the experimental design. The antibodies were diluted to the required concentration in 5% bovine serum albumin (BSA, Sigma-Aldrich) in PBS. Next day, sections were washed with PBS and further incubated with fluorochrome-conjugated secondary antibodies (Table 1) diluted in 1% BSA buffer for 2 h at room temperature. The DRG slices were then mounted with Vectashield plus DAPI, covered with coverslips and sealed with nail polish prior to imaging. Sections were kept in the dark, at 4°C and imaged with a Carl Zeiss LSM880 inverted confocal microscope (Carl Zeiss Ltd, Cambridge, UK) and processed with Zeiss ZEN imaging software. For each staining procedure, a buffer without primary antibody was used as a negative control and cells were detected by nuclear DAPI staining. Antibodies against STIM, Orai, NF200 and TRPV1 (Table 1) were explicitly characterised in previous studies. Specificity of JPH antibodies was tested in HEK293 cells transiently overexpressing flag-tagged JPH1-4, as well as with western blot in tissue homogenates known to express each of the proteins. To analyse the expression of STIM, Orai and JPH proteins, the number of positively and negatively stained neurons was determined. The mean fluorescence intensity of the area of the DRG section that was free of neuronal cell bodies was considered the threshold above which a neuron was considered ‘positive’. The cell diameter and mean intensity was calculated for each selected neuron. Cells were classed depending on the somatic diameter (presented as size-distribution histograms). Data are shown as numbers of positive neurons of different somatic sizes from the total number of neurons analysed. Cultured DRG neurons were fixed with 4% ice-cold PFA on glass coverslips for 20 min, the staining procedure was similar to that applied to DRG sections.

### Proximity ligation assay

PLA (Duolink, Sigma-Aldrich) was performed according to the manufacturer’s instructions and as described previously (Jin *et al*. 2013; Shah *et al*. 2020). In this approach, the proteins of interest within a cell/tissue are labelled with specific primary antibodies and then treated with PLA probes, which are secondary antibodies conjugated with short DNA oligos. If two proteins reside within less than 30–40 nm of each other, connector oligonucleotides facilitate the formation of a single-stranded DNA circle between the two secondary probes, a unique new DNA sequence is amplified, and a colour reaction is developed. DRG cultures were fixed with 4% ice-cold PFA for 20 min and permeabilised with buffer composed of 0.05% Tween 20 and 0.25% Triton X in PBS for 1 h at room temperature. Cells were then blocked for 30 min at 37°C (using Duolink blocking buffer) and incubated with primary antibodies (Table 1) for 12-16 h at 4°C. Following washing, the oligo-conjugated secondary antibodies (anti-mouse MINUS and anti-rabbit PLUS PLA probes, Sigma-Aldrich) were added for 1 h at 37°C. Ligation and amplification was performed according to the manufacturer’s instructions. Finally, the samples were washed and mounted with Duolink mounting medium with DAPI (Sigma-Aldrich). Imaging was performed using a Carl Zeiss LSM880 inverted confocal microscope and images processed by Zeiss ZEN software.

### Stochastic optical reconstruction microscopy (STORM)

The multicolour STORM was performed as described by us recently, using the same set of equipment and controls (Zhang *et al*. 2016; Shah *et al*. 2020). Briefly, images were acquired on a Nikon N-STORM super-resolution system (Nikon Instruments Inc., Melville, NY, USA), consisting of a Nikon Eclipse Ti inverted microscope and an astigmatic 3D lens placed in front of the EMCCD camera to allow the *Z*-coordinates to be most accurately determined. Two-colour STORM laser control was performed with non-overlapping activator dyes, Alexa 405 carboxylic acid (Invitrogen, Waltham, MA, USA, Cat. No. A30000) and Cy3 mono-reactive dye pack (GE Healthcare, Chicago, IL, USA, Cat. No. PA23001), conjugated to affinity-purified secondary antibodies from Jackson Immunoresearch (West Grove, PA, USA), along with the reporter dye Alexa Fluor 647 carboxylic acid (Invitrogen, Cat. No. A20006). The extent of co-localisation of STIM1 and JPH4 was determined with the same primary antibodies as in PLA experiments (Table 1). The activator and reporter fluorophores were conjugated in-house to an appropriate unlabelled secondary antibody. STORM imaging was performed in a freshly prepared imaging buffer that contained (in mm): 50 Tris (pH 8.0), 10 NaCl and 10% (w/v) glucose, with an oxygen-scavenging GLOX solution (0.5 mg/ml glucose oxidase (Sigma-Aldrich, St. Louis, MO, USA), 40 *μ*g/ml catalase (Sigma-Aldrich), and 10 mm cysteamine MEA (Sigma-Aldrich). MEA was prepared fresh as a 1 m stock solution in water. Acquisitions were made from between 2 and 3 different experiments for labelling and imaging. Images were rendered as 2D Gaussian fits of each localisation. The diameter of each point is representative of the localisation precision (larger diameter, less precise), as is intensity (more intense, more precise). Signal-noise thresholds were handled as peak height above the local background in the N-STORM software (Elements). A detected peak was set as the central pixel in a 5 × 5 pixel area, and the average intensity of the 4 corner pixels was subtracted from intensity of the central pixel. Using a 100× objective and 16 × 16 *μ*m pixel area of the iXon3 camera, this corresponds to a 0.8 × 0.8 *μ*m physical neighbourhood.

### Western blot

DRG neurons from Wistar rats 2–7 days old were seeded on 6-well plates. Cells were lysed 48 h post-seeding with lysis buffer (in mm): 50 HEPES, 150 NaCl 183.9 Na_3_VO_4_, 10 NaF, EDTA with 10% glycerol and 1% NP-40; supplemented with protease inhibitor. Lysates were homogenised and centrifuged for 10 min at 13,000 *g* at 4°C. DRG lysates were boiled for 5 min in SDS–polyacrylamide gel electrophoresis (SDS-PAGE) sample buffer (50 mm Tris HCl (pH 6.8), containing 5% 2-mercaptoethanol, 10% glycerol, and 1% SDS) and analysed by SDS-PAGE, followed by transfer onto a polyvinylidene difluoride membrane by electroblotting. The membranes were incubated in blocking buffer (Tris-buffered saline, TBS, containing 5% skimmed milk powder and 0.1% Tween 20 for 1 h, followed by incubation with primary antibodies (Table 1), diluted in the same buffer, at 4°C overnight. The membranes were washed in TBS containing 0.1% Tween 20 before 45 min incubation with an appropriate secondary antibody (horseradish peroxidase-conjugated anti-rabbit or anti-mouse secondary antibody). The bands were visualised using an enhanced chemiluminescence substrate (Pierce ECL WB Substrate and Super Signal West Femto, Thermo-Scientific). All samples were re-probed for *β*-actin as a loading control. The exposed films were processed using a Xograph machine, scanned and analysed using ImageJ software. All the western blot regagents were from Sigma-Aldrich, unless stated otherwise.

### *In vivo* siRNA knockdown

The *in vivo* JPH4 knock-down was performed as described earlier (Luo *et al*. 2005) by using the JPH4 siRNA2 oligonucleotide conjugated to cholesterol (conjugation was performed by RiboBio, Guangzhou, China). Cholesterol-conjugated JPH4 siRNA2 or scrambled control oligo were reconstituted in saline. The siRNA (200 nm, 10 *μ*l) or vehicle were injected intrathecally (i.t.) between the L5 and L6 dorsal spinous processes as described earlier (Huang *et al*. 2016; Zhang *et al*. 2019) under isoflurane anaesthesia. The treatment was repeated twice daily for 4 days. The pain tests were performed on the next day after the last injection. After behavioural testing, animals were humanly killed by overdose of isoflurane and confirmed by cervical dislocation; lumbar DRGs were extracted and analysed for the knockdown efficiency by RT-PCR.

### Behavioural assays

Adult male rats (body weight, 170−180 g, locally bred at the Hebei Medical University) were allowed to acclimate for at least 30 min in a transparent observation chamber before the experiment. For BK-induced pain assessment, animals received a 50-*μ*l intraplantar injection of BK (10 nmol/site) or saline into the right hindpaw. Animals were video-recorded for 30 min after the injection and protective (‘nocifensive’) behaviour was analysed as time spent licking, biting, lifting, and flinching the injected paw. The analyses were performed by an observer who was unaware of treatment allocations. For measurement of thermal sensitivity, the change in latency of hindpaw withdrawal in response to noxious heat was recorded using the Hargreaves’ plantar method (Ugo Basile, Gemonio, Italy); the heat source was set to 20% of the maximal intensity. Sensitivity to mechanical stimuli was assessed with the von Frey method, as described previously (Chaplan *et al*. 1994). Briefly, calibrated nylon filaments (von Frey hair, Stoelting, Wood Dale, IL, USA) with different bending forces were applied to the midplantar surface of the right hindpaw. The filaments were applied starting with the softest and continuing in ascending order of stiffness. A brisk withdrawal of the right hindlimb was considered a positive response. Each stimulus was applied five times with an interval of 5 s.

The threshold was recorded when three or more clear withdrawal responses within a set of five applications were observed with a given filament.

### Data analysis and statistics

Analysis of primary data and statistics were performed in Microsoft Excel, Origin Pro and Graphpad Prism. In fluorescence imaging experiments fura-2 ratios were normalised to *R* value at *t* = 0 (*R*_0_). Data from all cells were temporarily aligned for the onset of drug application.

All data are presented as mean ± standard deviation (SD) unless stated. Data were tested for normality using Shapiro-Wilk test. For normally distributed data sets, statistical significance was tested using Student’s paired or unpaired *t* test, or ANOVA with Tukey’s *post hoc* test. For non-normal data, the Wilcoxon signed rank test (for paired data) or Mann-Whitney test (for unpaired data) were used for single-group comparisons and Kruskal-Wallis ANOVA with Mann-Whitney *post hoc* test for multiple group comparisons. Differences between means were considered significant at *P* < 0.05. Statistical parameters for each data set presented in this study are listed in the Statistical Summary Document in Supporting information. For datasets with n > 30, the raw datasets are provided in the Raw Datasets Document in Supporting information.

In immunohistochemical experiments the cell diameter and mean fluorescence intensity were calculated for each selected neuron; proportions of neurons expressing proteins of interest were analysed using Fisher’s exact test.

Co-localisation analysis was performed using the JACoP plugin (Bolte & Cordelieres, 2006) for ImageJ (Fiji). In the confocal images of DRG neurons, layers were split into green and red channels. These were loaded into the JACoP analysis and intensity correlation analysis (ICA) (Li *et al*. 2004) was performed. The method assumes that the overall difference between pixels intensities and the mean intensity of a single channel is equal to zero: $\sum n_{\text{pixels}}(A-a)=0$ and $\sum n_{\text{pixels}}(B-b)=0$; where *A* and *B* are the current pixel intensities and *a* and *b* are the current channel mean intensities. Thus, the product of the two equalities should tend to zero. For a co-localising pixel, this product should be positive because each difference from the mean is of the same sign. The differences in intensities between both channels are scaled down by fitting the histogram of both images to a 0–1 scale. In the ICA graph the normalised intensities (from 0 to 1) are shown as a function of the product (*Ai*− *a*)(*Bi*− *b*) for each channel. In the case of co-localisation, the product (*Ai* −*a*)(*Bi*− *b*) is positive and therefore the dot cloud is mostly concentrated on the right side of *x* = 0 line. Thresholds were set in JACoP. Once the analysis had been completed, data were saved as a txt file and exported into Excel for graphical representation.

### Cluster size and localisation proximity analysis of STORM data

Unfiltered STORM localisation data were exported as molecular list text files from Nikon-Elements and were analysed with in-house software incorporating a density-based spatial clustering of applications with noise (DBSCAN) (Zhang *et al*. 2016). A dense region or cluster was defined as localisations within a directly reachable radius proximity (epsilon) from a criterion minimum number of other core localisations (MinPts). Density-reachable points were localisations that were within the epsilon radius of a single core point and thus considered part of the cluster. Localisations considered to be noise were points that were not within the epsilon distance of any core points of a cluster. We derived the appropriate epsilon parameter using the nearest-neighbour plot from single-dye labelled controls; cluster detection was determined for epsilon between 20 and 80 nm, which were the nearest-neighbour localisation distances representing 95% of area under the curve. DBSCAN parameters were verified by measuring goodness of fit to Gaussian distribution with cluster population data from single-dye labelled controls, and set for distance of directly reachable points at 50 nm (epsilon) and five minimum points (MinPts). These parameters were found to be the most stringent possible that also reliably fitted the control data. For cluster detection, each localisation was assessed based on its corrected *X* and corrected *Y* 2D spatial coordinates, and the associated activator dye was tracked throughout analysis. Detected clusters were tabulated by the composition of resident activator dyes contributing to the total neighbourhood of localisations for that cluster. Clusters were categorised based on activator dye composition as Alexa 405 only, Cy3 only, or Alexa 405 + Cy3 according to the dye conjugated to each antibody label. Cluster radius size (nm) data were placed in probability distribution histograms with bin size of 5 nm. Based on the observation that a majority of cluster distributions displayed positive skew, distributions were fitted by the generalised extreme value distribution function: $\text{f}(x)={\text{e}}^{-(x-1+{\text{e}}^{x}-x)}$. Cluster size values were reported as means ± SD. In addition, the percentage of clusters belonging to a labelling category out of total clusters was compared. Population statistics for each cluster category were derived from 7 to 8 cells per staining and imaging condition.

## Results

### Expression of junctophilins in sensory neurons

**Figure 1:**
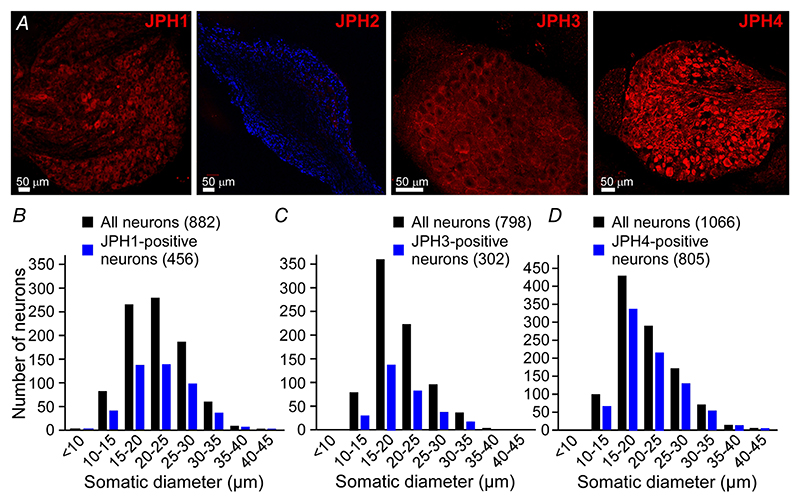
Expression of junctophilin isoforms in the DRG *A*, confocal micrographs for immunostaining of DRG sections with antibodies against JPH1-4, as indicated. DAPI staining is included in the JPH2 micrograph to depict DRG section. Scale bars = 50 *μ*m. *B*–*D*, the somatic diameters of neurons positive for JPH1 (*B*), JPH3 (*C*) or JPH4 (*D*) were measured and plotted as size-distribution histograms alongside the total number of neurons (black). Data are shown as the number of immunofluorescence-positive neurons within each somatic size-band out of the total neurons analysed; cumulative data from 6 individual DRG sections for each protein analysed. [Colour figure can be viewed at wileyonlinelibrary.com]

**Figure 2:**
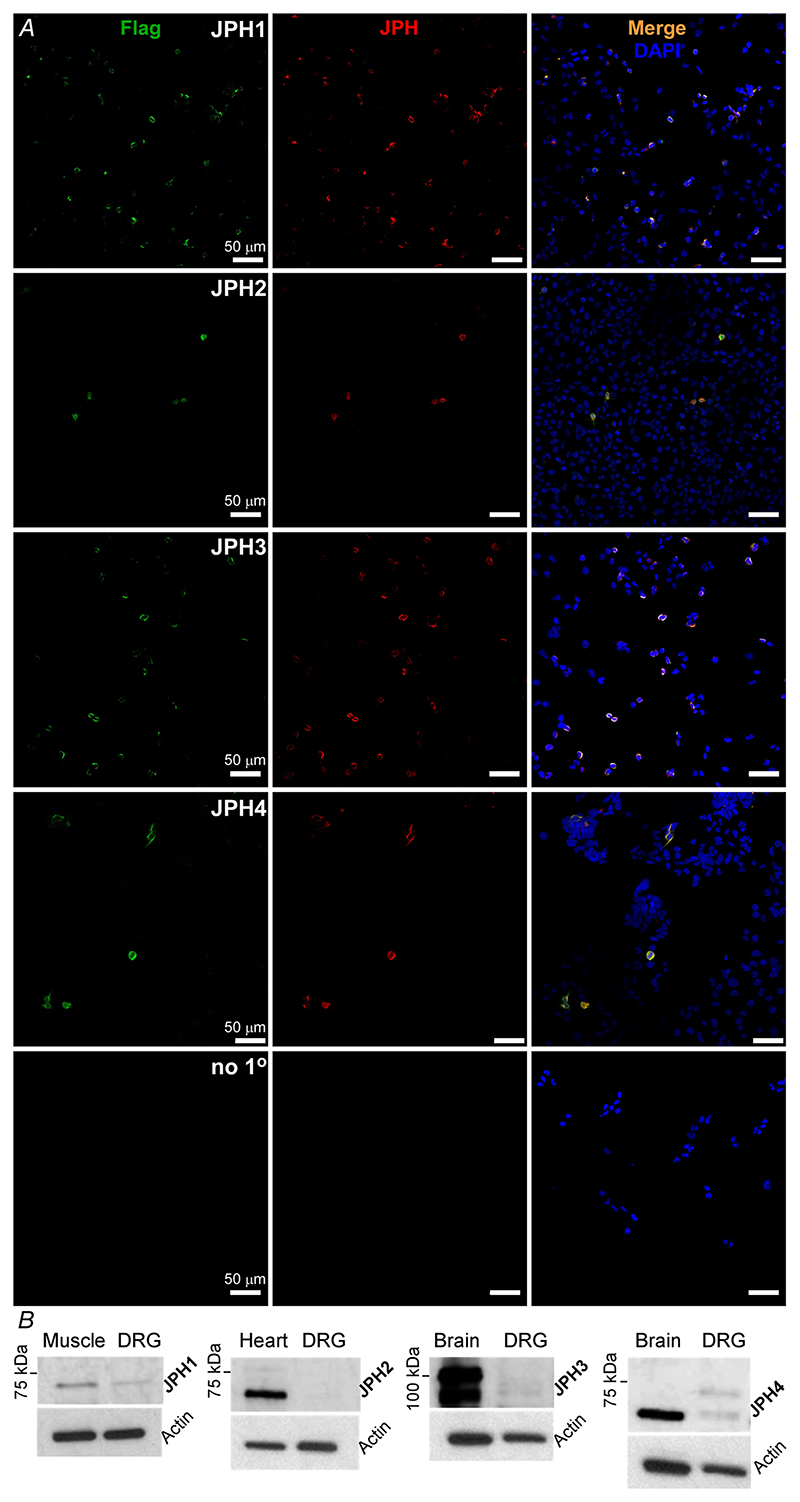
Validation of junctophilin antibody specificity and tissue expression patterns *A*, HEK293 cells were individually transfected with flag-tagged JPH1-4 encoding plasmids (see Methods) and immunostained with anti-flag and anti JPH1-4 antibodies (Table 1). Scale bars = 50 *μ*m. *B*, western blot analyses of the JPH family members in DRG and positive control tissue lysates (skeletal muscle for JPH1, heart for JPH2 and brain for JPH3 and 4). These are representative results of 6 individual experiments for each JPH isoform. [Colour figure can be viewed at wileyonlinelibrary.com]

ER-PM junctions are maintained by junctional protein anchors and of these, the junctophilin family is perhaps one of the best understood (Takeshima *et al*. 2015). Yet to the best of our knowledge the expression profile and potential function of junctophilins in sensory neurons is hitherto unreported. Thus, we performed immunohistochemistry and western blot experiments to investigate expression of junctophilins in DRG (Figs 1 and 2). The specificity of antibodies against JPH1-4 was tested in HEK293 cells individually transfected with flag-tagged JPH1-4 (Fig. 2A). For each individual transfection the labelling with anti-flag and individual anti-JPHx antibody revealed an exact match. Both immunohistochemistry (Fig. 1A) and western blot (Fig. 2B) revealed no detectable levels of JPH2 in DRG. JPH1, JPH3 and JPH4 were found in 52% (456/882), 38% (302/798) and 76% (805/1066) of DRG neurons analysed by immunohistochemistry, respectively. JPH4 was expressed in a significantly higher proportion of neurons, compared to JPH1 (*P* < 0.00001) and JPH3 (*P* < 0.00001, Fisher’s exact test). Size distribution analysis of neurons displaying immunoreactivity for JPH1, JPH3 and JPH4 did not reveal a bias towards neurons of a particular size (Figs 1B–D). Western blot analysis (Fig. 2B) confirmed the presence of JPH1, 3 and 4, but not JPH2 in DRG, while all JPH proteins were detected in their specific control tissues. Interestingly, the JPH4 antibody revealed a double band in DRG lysate, one of which was of the predicted molecular weight (66 kDa), but only a lighter band was detected in the brain sample, suggesting the existence of at least two forms of this protein (e.g. alternatively spliced gene products or a post-transcriptional modification).

**Figure 3:**
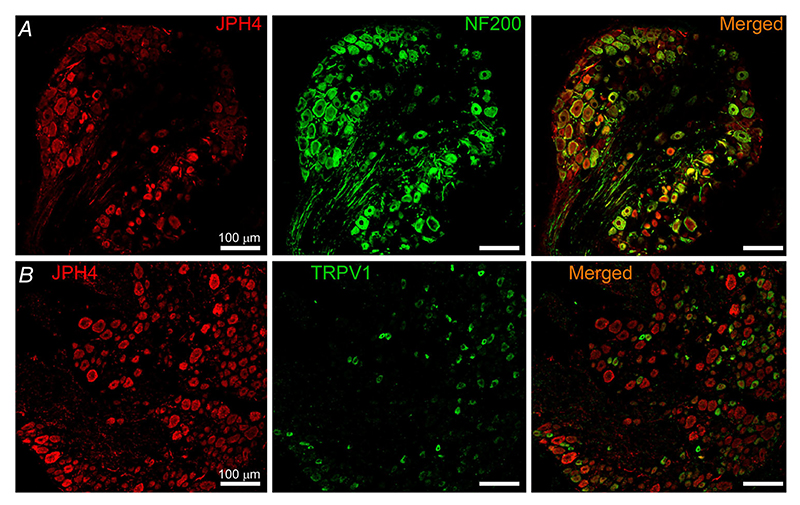
Expression of JPH4 in subpopulations of DRG neurons Confocal micrographs for co-immunostaining of DRG sections with antibodies against JPH4 and neurofilament 200 (NF200, *A*) and TRPV1 (*B*). Scale bars = 50 *μ*m. [Colour figure can be viewed at wileyonlinelibrary.com]

As JPH4 was found to be expressed in the larger proportion of DRG neurons, we focused on this isoform in subsequent studies. Co-staining of JPH4 with neuronal markers NF200 (myelinated A*β* and A*δ* neurons) and TRPV1 (mostly polymodal nociceptors) revealed that 75% of the NF200-positive and 84% of the TRPV1-positive neurons also displayed JPH4 immunoreactivity (Fig. 3).

**Figure 4:**
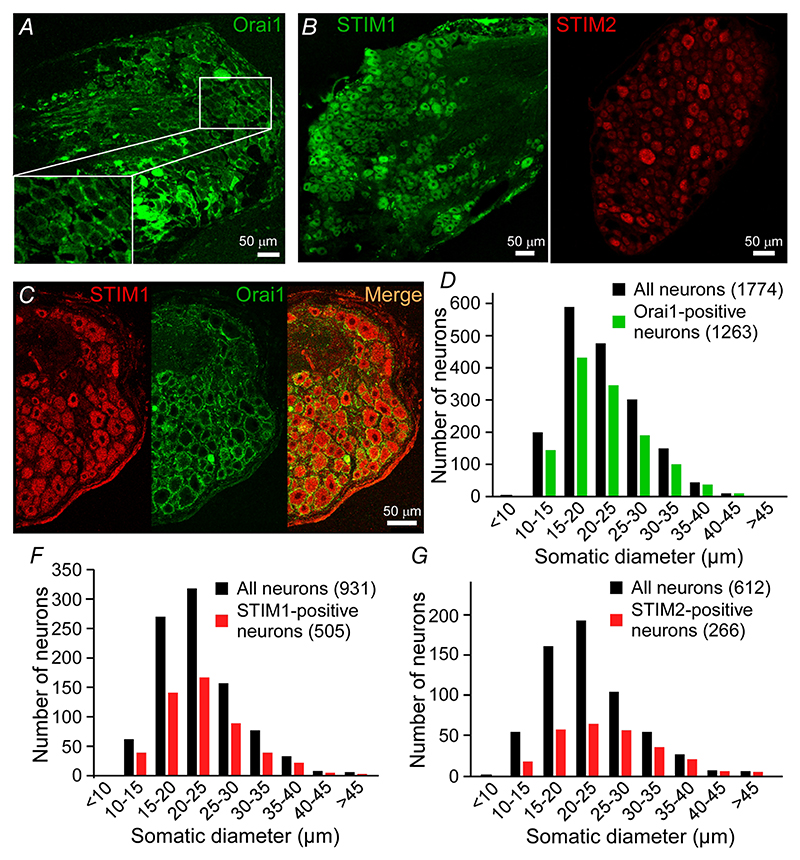
Expression of Orai1 and STIM isoforms in the DRG Confocal micrographs for immunostaining of DRG sections with antibodies against Orai1 (*A*), STIM1 and STIM2 and co-immunostaining of Orai1 and STIM1 (*C*). Scale bars = 50 *μ*m. *D*–*G*, the somatic diameters of neurons positive for Orai1 (*D*), STIM1 (*F*) or STIM2 (*G*) were measured and plotted as size-distribution histograms alongside the total number of neurons (black). Data are shown as the number of immunofluorescence-positive neurons within each somatic size-band out of the total neurons analysed; cumulative data from 10 individual DRG sections for Orai1 and from 5 individual sections for the other proteins are shown. [Colour figure can be viewed at wileyonlinelibrary.com]

We hypothesised that JPH4 may facilitate ER-PM signalling processes, such as SOCE. Thus, next we thought to confirm presence in DRG neurons of the major proteins constituting the SOCE complex: STIM1-2 and Orai1. Immunohistochemical staining of DRG sections demonstrated that STIM1, STIM2 and Orai1 were expressed in DRG neurons (Fig. 4). Orai1 was expressed in 72% of all neurons tested (1263/1774); immunoreactivity had obvious PM localisation (Fig. 4A). Size distribution analysis suggested that there was no evident preference of Orai1 expression in neurons of a particular size, suggesting broad expression among neurons of different sensory modalities (Fig. 4D). We also tested Orai2 and Orai3 expression; the Orai2 signal was mainly localised to the nucleus (not shown) and was therefore not analysed. Orai3 immunoreactivity was generally weak (not shown).

**Figure 5:**
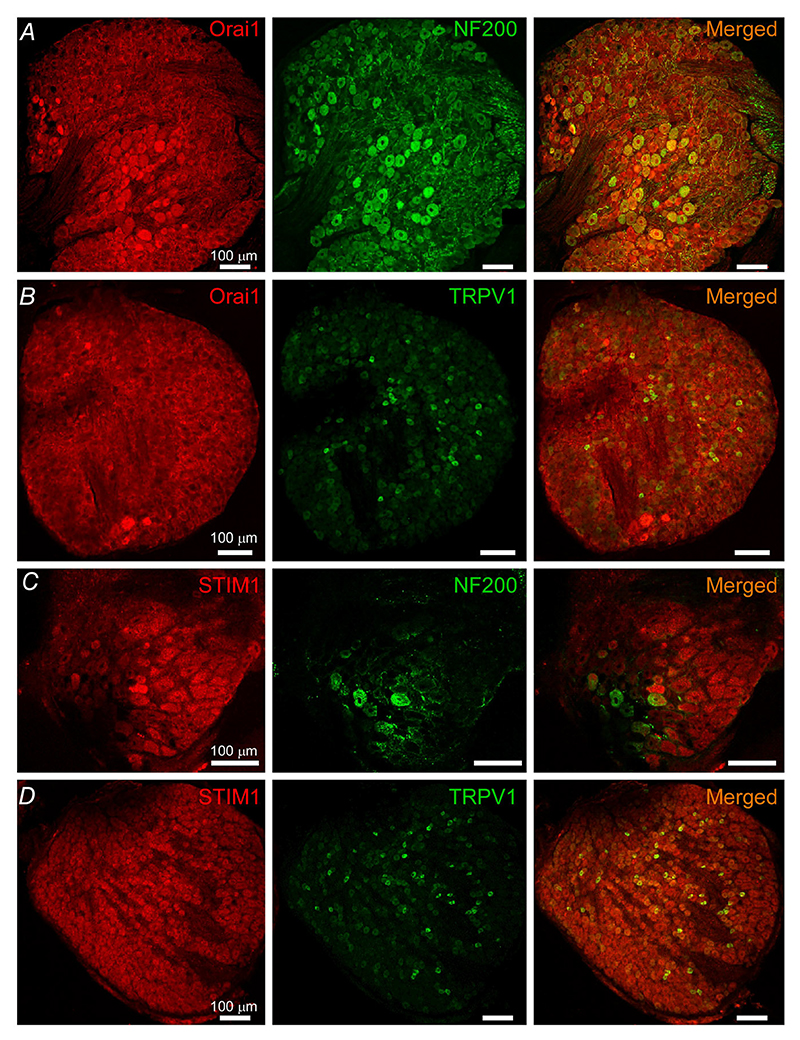
Expression of Orai1 and STIM1 in subpopulations of DRG neurons Confocal micrographs for co-immunostaining of DRG sections with antibodies against Orai1 and neurofilament 200 (NF200, *A*), Orai1 and TRPV1 (*B*), STIM1 and NF200 (*C*), STIM1 and TRPV1 (*D*). Scale bars = 50 *μ*m. [Colour figure can be viewed at wileyonlinelibrary.com]

STIM1 was expressed in 54% (505/931) of all neurons analysed; the immunoreactivity was also distributed evenly across the neurons of different sensory modalities (Fig. 4B, left panel and *F*). There was a large overlap between STIM1 and Ora1 expression patterns (Fig. 4C; also see below). STIM2 immunoreactivity was found in 43% (266/612) of all DRG neurons analysed (Fig. 4B, right panel), an incidence which was significantly lower than that of STIM1 (*P* < 0.01; Fisher’s exact test). There was a bias towards the neurons of larger diameter amongst STIM2-positive neurons (Fig. 4G). Co-staining of Orai1 (Fig. 5A and B) or STIM1 (Fig. 5C and D) with NF200 or TRPV1 confirmed ubiquitous distribution of both SOCE complex proteins in DRG.

These analyses identified STIM1 and Orai1 as the most ubiquitously expressed SOCE complex proteins in DRG neurons. In addition, Orai2 was found not to be necessary for SOCE in DRG neurons (Wei *et al*. 2017). Thus, we concluded that the ‘classic’ Orai1-STIM1 complex is the most abundant SOCE correlate in the majority of DRG neurons. We therefore focused on this complex in subsequent experiments. However, a contribution of other subunits to SOCE in DRG neurons cannot be excluded.

**Figure 6:**
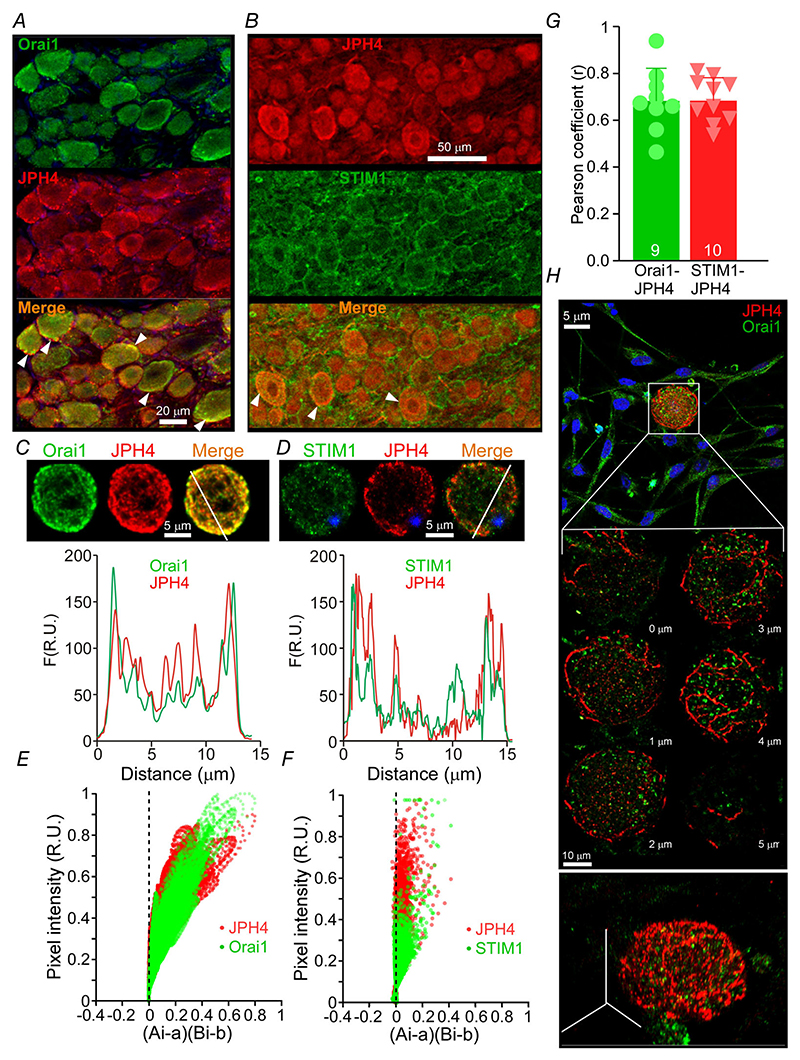
Intracellular localisation of JPH4 in DRG neuron somata *A* and *B*, co-immunostaining of JPH4 with Orai1 (*A*) or STIM1 (*B*) in DRG. *C* and *D*, Airyscan images of JPH4-Orai1 or JPH4-STIM1 (*D*) co-labelled DRG neuron in culture; lower panels show the fluorescence intensity line scans displaying overlapping intensity maxima for both proteins at the PM. R.U., relative units. *E* and *F*, intensity correlation analysis (ICA; see Methods) of the entire cell area from images shown in panels *C* and *D*; *x*-axis reflects the covariance and the *y*-axis reflects the intensity distribution of the two fluorescence signals. Position of the dots on the right side of *x* = 0 line indicates co-localisation. *G*, co-localisation analysis for Orai1 (*n* = 9 neurons) or STIM1 (*n*= 10 neurons) and JPH4 using the Pearson correlation. Scatter plot shows Pearson coefficients (*r*) for individual neurons; bars represent means; error bars represent SD. *H*, Airyscan image (*Z*-stack of 1 *μ*m optical sections) of a cultured DRG neuron revealing a specific tubular localisation of JPH4 (red) near the plasma membrane. Lower panel is a 3D representation of the same neuron. [Colour figure can be viewed at wileyonlinelibrary.com]

Co-staining of JPH4 with either Orai1 (Fig. 6A) or STIM1 (Fig. 6B) revealed a high degree of co-expression of these proteins in neurons. Figure 6C and D depicts small-diameter DRG neurons in culture imaged using an improved-resolution Airyscan method (LSM880, Zeiss).

Orai1, JPH4 and STIM1 all displayed fluorescence maxima at the PM; the Orai1/JPH4 (Fig. 6C) and STIM1/JPH4 (Fig. 6D) PM maxima significantly overlapped, suggesting close proximity. Co-localisation of JPH4 with Orai1 and STIM1 was further analysed using intensity correlation analysis (ICA, see Methods; Fig. 6E and F) and the Pearson correlation method (Fig. 6G), both revealing a high degree of co-localisation. In many individual DRG neuron somata, JPH4 immunoreactivity displayed a striking tubular appearance with a preferred distribution towards the PM, rather than being randomly diffused in the cytoplasm; an example of such a JPH4-positive tubular network is given in Fig. 6H. These observations suggest that JPH4 might be present at the ER-PM junctional nanodomains of DRG neurons.

### Role of JPH4 in junctional signal transduction in DRG neurons

**Figure 7:**
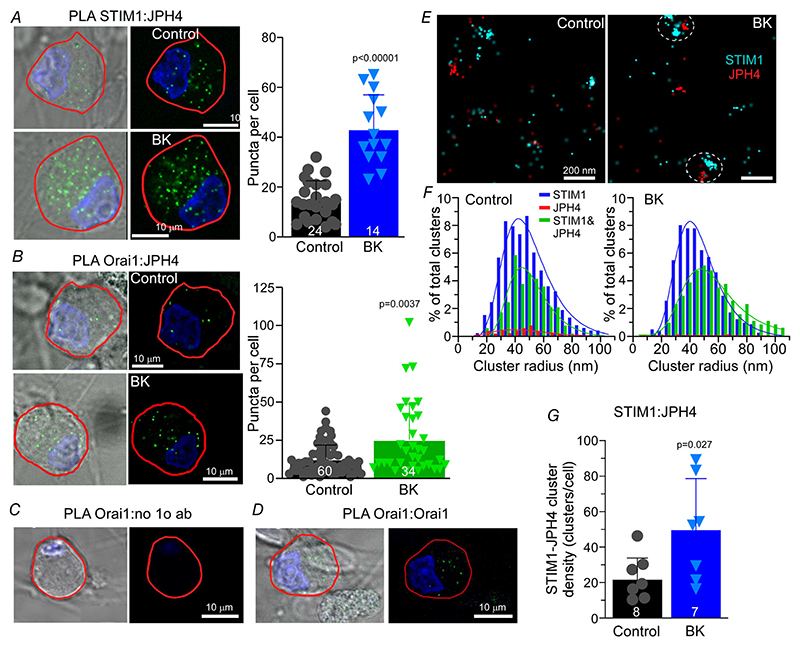
JPH4 interacts with the SOCE complex in DRG neurons *A*, proximity ligation assay (PLA) probing JPH4 and STIM1 proximity in small diameter DRG neurons. Cells were left untreated (Control) or treated with 250 nM BK for 15 min in Ca^2+^ free solution to induce store depletion. Scatter plot shows number of PLA puncta for individual neurons; bars represent means; error bars represent SD; *n* = 24 neurons (control), 14 neurons (BK); *N* = 6 independent experiments. Scale bars =10 *μ*m. *B*, experiments similar to that shown in panel *A* but for Orai1:JPH4 proximity (*n* = 60 neurons (control), 34 neurons (BK); *N* = 6 independent experiments). Labelling and experimental conditions as in *A. C* and *D*, PLA protocol validation: *C*, negative control; only one primary antibody against Orai1 was used in combination with both secondary PLA probes. *D*, positive control; anti-Orai1 mouse and anti-Orai1 rabbit antibodies were paired to the corresponding secondary PLA probes. Scale bars = 10 *μ*m. *E*, representative stochastic optical reconstruction microscopy (STORM; see Methods) images from control or BK-treated (250 nM BK for 15 min in Ca^2+^ free solution) DRG neurons, double-labelled for STIM1 using dye-pairs of AF405/647 (cyan centroids) and JPH4 using dye-pairs of Cy3/647 (red centroids); scale bars = 200 nm. *F*, comparison of distributions of clusters representing double-labelled STIM1-JPH4 under control and BK-stimulation conditions, respectively (see Methods). *G*, quantification of cluster density in experiments as those shown in panel *E*, scatter plot shows number of STIM1-JPH4 clusters for individual neurons; bars represent means; error bars represent SD; *n* = 7 neurons (control), 7 neurons (BK); *N* = 3 independent experiments. [Colour figure can be viewed at wileyonlinelibrary.com]

Since clustering of STIM1 with Orai1 and SOCE complex formation is a signature response to GPCR-induced ER store depletion (Qiu & Lewis, 2019), we used proximity ligation assays (PLA, an ‘in situ proteomics’ approach; Soderberg *et al*. 2006; Weibrecht *et al*. 2010) to test if JPH4 is involved in this process in DRG. This method allows specific detection of closely associated proteins *in situ*. Bright fluorescent PLA puncta of 0.5–1 *μ*m diameter are formed only if the proximity of the proteins of interest is no more than 30–40 nm (Soderberg *et al*. 2006; Weibrecht *et al*. 2010; see Methods). The proximity between STIM1 and JPH4 (Fig. 7A), and Orai1 and JPH4 (Fig. 7B) was significantly increased following ER Ca^2+^ store depletion with BK treatment (250 nm; 15 min in Ca^2+^-free extracellular solution). This suggests that while some level of clustering between JPH4, STIM1 and Orai1 might already exist at resting conditions, the clustering is significantly increased in response to ER Ca^2+^ release. This, in turn, might suggest that JPH4 facilitates SOCE by either promoting ER-PM proximity or by directly facilitating STIM1-Orai1 interactions. Specificity of PLA has been confirmed by either omitting one of the primary antibodies, while keeping both secondary PLA probes (negative control; Fig. 7C) or by using two different primary antibodies against the same protein (positive control, Fig. 7D).

To further confirm the interaction of STIM1 and JPH4 in response to the BK-induced ER store depletion, we used a dual-colour super-resolution STORM imaging approach developed by us specifically to probe protein interaction in sensory neurons (Zhang *et al*. 2016; Shah *et al*. 2020). Control or BK-treated (250 nm; 15 min in Ca^2+^-free extracellular solution) DRG cultures were fixed and labelled with primary antibodies for STIM1 and JPH4, followed staining by secondary antibodies conjugated to photoswitchable fluorophores (see Methods). Using near-TIRF, oblique-angle illumination, nanodomain proximity of STIM1 and JPH4 has been observed, denoted by clustered STORM co-localisations (Fig. 7E–G). In the BK-treated neurons, there were significantly more STIM1-JPH4 clusters compared to control (Fig. 7G), consistent with the PLA experiments. On examination of the population distribution of detected clusters, there was botha right-shift in cluster size (Fig. 7F) and a significantly increased percentage of STIM1-JPH4 interactions (Fig. 7G) in BK-treated DRGs compared to control, but there were no significant differences between conditions in the STIM1-only or JPH4-only clusters.

**Figure 8:**
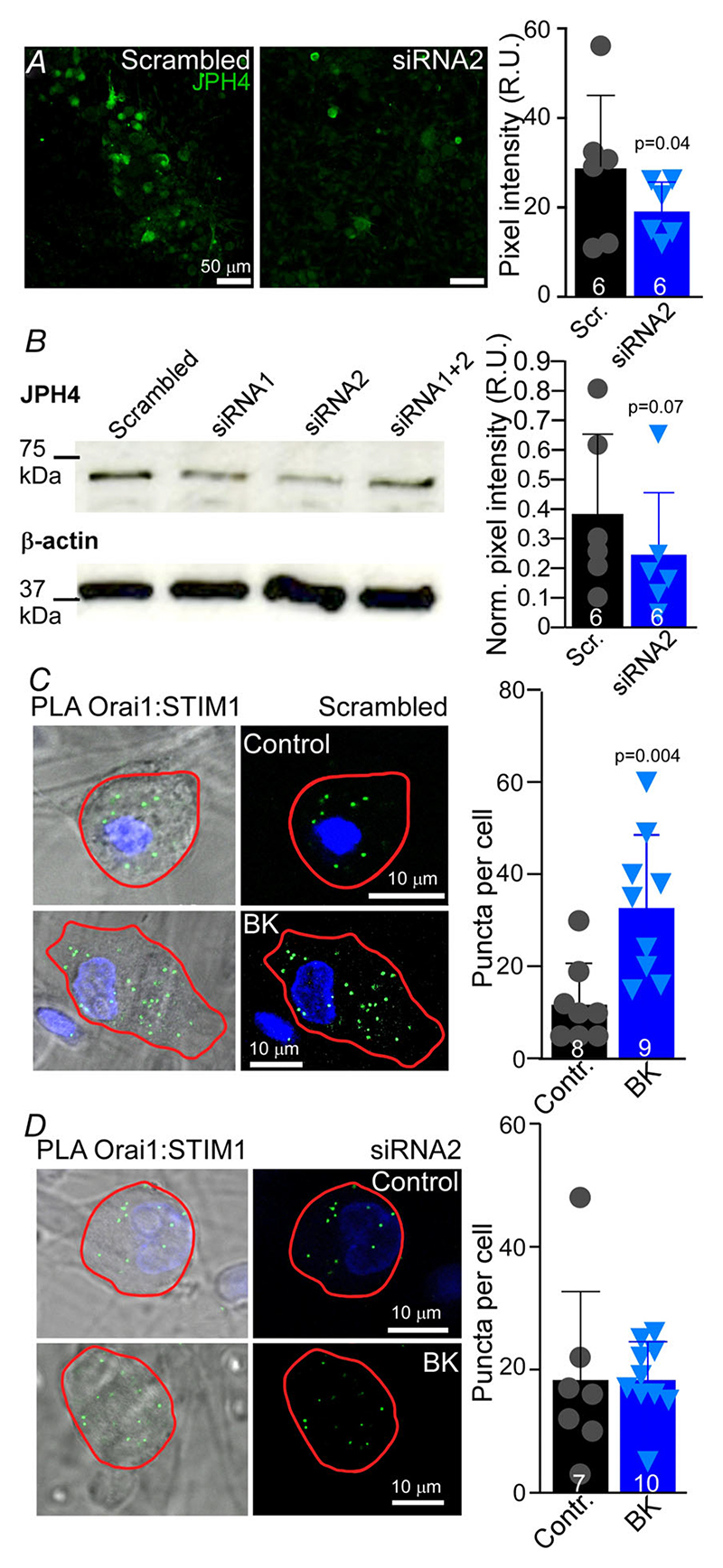
Knockdown of JPH4 in DRG neurons disrupts STIM1:Orai1 clustering *A*, assay of JPH4 immunofluorescence in DRG culture transfected with scrambled oligo or JPH4-siRNA2. The data are summarised in the scatter plot on the right; bars represent means; error bars represent SD; *n* = 6 for both data sets; R.U., relative units. *B*, western blot analysis of JPH4 abundance in DRG lysates from cultures transfected with JPH4-siRNA1, JPH4-siRNA2 or scrambled oligo. The data are summarised in the scatter plot on the right; bars represent means; error bars represent SD; *n* = 6 for both data sets. *C* and *D*, analysis of fluorescent PLA signals produced by clustering of Orai1 and STIM1 in cultured DRG neurons transfected with scrambled oligo (*C*) or JPH4-siRNA2 (*D*). Cells were left untreated (scrambled oligo: *n* = 8, *N* = 4; JPH4 siRNA2: *n* = 9, *N* = 5) or treated with 250 nM BK for 15 min in Ca^2+^-free solution (scrambled oligo: *n* = 7, *N* = 5; JPH4 siRNA2: *n* = 10, *N* = 5) to induce store depletion. Quantification of PLA puncta density is shown in the scatter plots; bars represent means; error bars represent SD. [Colour figure can be viewed at wileyonlinelibrary.com]

Next, we tested if JPH4 knockdown in DRG neurons would affect STIM1-Orai1 interaction. Two different siRNA targeting JPH4 were used at various concentrations, separately or in combination to optimise the protocol. The siRNA2 (see Methods) demonstrated the highest efficacy of reducing JPH4 protein expression in DRG neurons 48 h post transfection, as tested using immunohistochemistry and western blot analysis (Fig. 8A and B). PLA experiments testing the interaction between Orai1 and STIM1 were performed on DRG neurons transfected with JPH4 siRNA2 or scrambled oligo. Treatment of DRG neurons transfected with scrambled oligo with BK (250 nm; 15 min in Ca^2+^-free extracellular solution) significantly increased Orai1-STIM1 interaction indicating SOCE complex formation. This was revealed as a significant increase in PLA puncta number, compared to vehicle-treated controls (Fig. 8C). Strikingly, no significant difference between the number of puncta in BK-treated and vehicle-treated neurons was detected when JPH4 was silenced (Fig. 8D), suggesting that Orai1-STIM1 interaction was attenuated.

**Figure 9:**
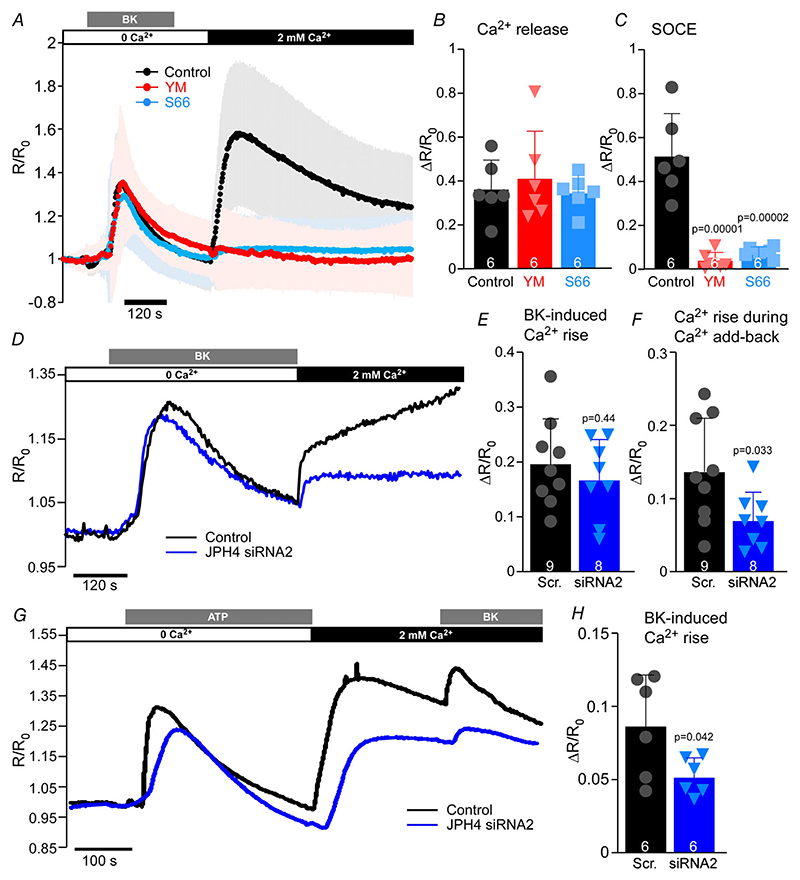
Knockdown of JPH4 in DRG neurons impairs SOCE *A*, fura-2 Ca^2+^ imaging traces of the BK-induced (250 nM) store depletion and Ca^2+^ add-back-induced SOCE in DRG neurons of control neurons (black) and neurons pre-treated with 1 *μ*M YM58483 (red) or 3 *μ*M Synta66 (blue) for 1 h; the inhibitors were also present in extracellular solutions throughout the experiment. Shown is mean normalised *F*_340_/*F*_380_ fluorescence intensity ratio (*R/R*_0_ ± SD; *n* =99 neurons (control), 104 neurons (YM58483) and 71 neurons (Synta66); *N* = 6 independent experiments for each group). Scatter plots summarise the effect of YM58483 and Synta66 on the peak BK-induced Ca^2+^ release (*B*) and Ca^2+^ add-back-induced SOCE (*C*). Scatter plots represent *ΔR/R*_0_ means for each independent experiment; bars represent overall means; error bars represent SD. *D*, representative example of fura-2 Ca^2+^imaging recording of typical store depletion/Ca^2+^ add-back experiments in control (scrambled oligo) and JPH4-siRNA2 transfected DRG neurons. *E* and *F*, summary data for experiments as these shown in *D*; the effect of JPH4 KD on the amplitude of the BK-induced Ca^2+^ transients (*E*) and SOCE during the Ca^2+^ re-addition phase (*F*). Scatter plots represent *ΔR/R*_0_ values for individual neurons; bars represent means; error bars represent SD; scrambled oligo: *n* = 9, *N* = 3; JPH4 siRNA2: *n* = 8, *N* = 3. *G*, representative example of fura-2 Ca^2+^ imaging recording in control (scrambled oligo) and JPH4-siRNA2 transfected DRG neurons. First store depletion was induced by ATP (30 *μ*M) in the Ca^2+^-free buffer; Ca^2+^ was then added back to the extracellular solution and secondary IP_3_-dependent store depletion with BK (250 nM) was performed. *H*, summary data for experiments as these shown in *G*: the effect of JPH4 KD on the amplitude of the BK-induced Ca^2+^ transients during the Ca^2+^ re-addition phase. Scatter plots represent *ΔR/R*_0_ values for individual neurons; bars represent means; error bars represent SD; scrambled oligo: *n* = 6, *N* = 3; JPH4 siRNA2: *n* = 6, *N* = 3. [Colour figure can be viewed at wileyonlinelibrary.com]

Next, we set out to test how JPH4 knockdown would affect BK-induced Ca^2+^ signalling in DRG neurons. First, we established a measurement of the SOCE in cultured, small-diameter DRG neurons (mostly nociceptors). We performed ratiometric Ca^2+^ imaging on DRG neurons loaded with fura-2AM; SOCE was induced with 250 nm BK in Ca^2+^-free extracellular solution for 4 min, followed by re-addition of 2 mm Ca^2+^ to the extracellular solution to facilitate *I*_CRAC_. To confirm the identity of the Ca^2+^ transient induced by the extracellular Ca^2+^ add-back we used two SOCE inhibitors: a widely-used pyrazole derivative, YM58483 (BTP2) (Ishikawa *et al*. 2003), and a newer, selective inhibitor, Synta66 (Tian *et al*. 2016) (Fig. 9A). Either YM58483 (1 *μ*m) or Synta66 (3 *μ*m) were applied 1 h prior to Ca^2+^ imaging, during fura-2AM loading and were also present throughout the recording. Only neurons responsive to BK were included in the analysis; the BK-responsive DRG neurons under our experimental conditions are largely TRPV1-positive poly-modal nociceptors (Liu *et al*. 2010). Neither YM58483 nor Synta66 significantly altered the Ca^2+^ release from the ER induced by BK; however, both drugs almost entirely abolished Ca^2+^ influx during the Ca^2+^ add-back phase of the experiment (Fig. 9B). YM58483 and Synta66 reduced the amplitude of the Ca^2+^ add-back transient by 93% (*P* = 0.00001; *n* = 104, *N* = 6) and by 86.5% (*P* = 0.00002; *n* = 71, *N* = 6), respectively.

In the next series of experiments we compared the amplitudes of BK-induced and Ca^2+^ add-back-induced Ca^2+^ transients in DRG neurons transfected with either JPH4 siRNA2 or scrambled oligo using the experimental protocol presented in the Fig. 9B. Silencing JPH4 in DRG did not significantly affect the amplitude of the BK-induced Ca^2+^ transient (Fig. 9D and E) but reduced the SOCE (Ca^2+^ transient during the Ca^2+^ add-back) by 45% (*n* = 8; *P* = 0.033; Fig. 9D and F). These results suggest that JPH4 might be involved in facilitating SOCE; yet the basal ER Ca^2+^ levels could be maintained in unstimulated cells even with such partial SOCE impairment.

We then reasoned that while ER Ca^2+^ stores can be maintained in unstimulated neurons even with JPH4 knocked down, impaired SOCE in JPH4-defficient neurons could impede recurrent GPCR signalling as ER stores would not replenish fully in such conditions. To test this hypothesis, we designed an experimental protocol where two rounds of store depletion were applied in sequence. To avoid the desensitisation and tachyphylaxis common for the GPCR, including B_2_R (Bawolak *et al*. 2009), DRG neurons were treated with two different GPCR-agonists. Thus, cultured cells were first stimulated with 30 *μ*m ATP (in Ca^2+^-free extracellular solution) to activate endogenous G_q/11_-coupled P2Y receptors expressed in DRG neurons (Gerevich & Illes, 2004). Following the Ca^2+^ add-back phase, BK was applied to trigger a second round of the IP_3_-dependent store depletion, this time by activating B_2_R. It has to be pointed out that even though DRG neurons also express ionotropic ATP-activated P2X_2_/P2X_3_ receptors (Gerevich & Illes, 2004), our experimental protocol excluded the contribution of these ion channels to the ATP-induced [Ca^2+^]_i_ transients, since ATP was applied in Ca^2+^-free extracellular solution (in line with previous protocols). Figure 9G and H shows that the secondary (BK-induced)

Ca^2+^ release from the stores was indeed reduced by 40% (*n* = 6; *P* = 0.042) in JPH4-silenced neurons compared to scrambled oligo controls. The ATP-induced Ca^2+^ transients were also somewhat smaller in JPH4-silenced neurons, but this difference did not reach significance. Taken together, the experiments presented in Figs 8 and 9 demonstrate that (i) JPH4 is necessary for the ER store depletion-mediated Orai1-STIM1 clustering in DRG neurons, (ii) JPH4 knockdown impairs SOCE, and (iii) JPH4 knockdown reduces the capacity of the ER Ca^2+^ stores to sustain recurrent GPCR-mediated Ca^2+^ signalling.

### Knockdown of JPH4 *in vivo* shortens the duration of inflammatory nociception

**Figure 10:**
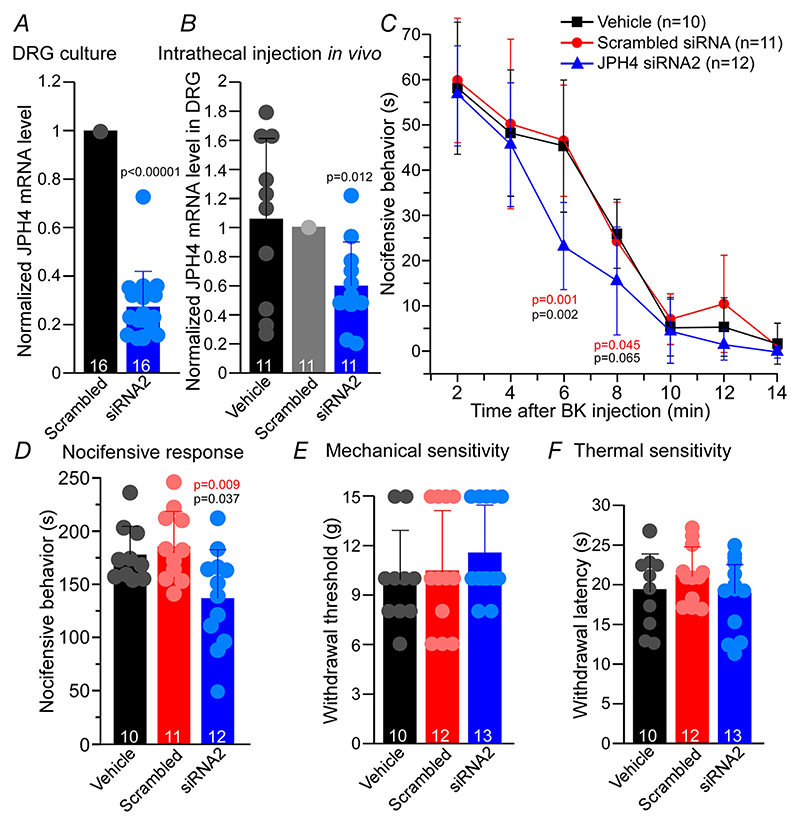
Knockdown of JPH4 in DRG *in vivo* attenuates BK-induced pain *A* and *B*, efficiency of the JPH4 knockdown (KD) using the cholesterol-conjugated JPH4-siRNA2 as tested by RT-PCR. *A*, application of cholesterol-conjugated JPH4-siRNA2 or scrambled oligo (200 nM) to dissociated DRG neurons in culture. Scatter plots represent individual transcript levels normalised to scrambled oligo control; bars represent means; error bars represent SD; *n* = 16. *B*, intrathecal injections (2 pmol/site; twice a day for 4 days) of JPH4-siRNA2, scrambled oligo or vehicle *in vivo*. L5-L6 DRG were analysed for JPH4 expression using RT-PCR. Scatter plots represent transcript levels in L5-L6 DRGs from individual animals, normalised to scrambled oligo control; bars represent means; error bars represent SD; *n*= 11; *P* values represent differences for JPH4 siRNA-injected animals compared to scrambled oligo. *C*, intrathecal JPH4 KD attenuated BK-induced nocifensive behaviour. BK (10 nmol/site; 50 *μ*l) was injected into the right hindpaw and the time spent licking, biting and flinching the injected paw was recorded and plotted in 2-min intervals. *P* values in red represent differences for JPH4 siRNA-injected animals compared to scrambled oligo; *P* values in black represent differences for JPH4 siRNA-injected animals compared to vehicle (vehicle: *n*= 10; scrambled oligo: *n* = 11; JPH4-siRNA2: *n* = 12). *D*, total time of nocifensive behaviour over 30 min observation period in the same animals analysed in panel *C*. Scatter plots represent total times of nocifensive behaviour for individual animals; bars represent means; error bars represent SD. *P* value in red represents difference for JPH4 siRNA-injected animals compared to scrambled oligo; *P* value in black represent difference for JPH4 siRNA-injected animals compared to vehicle (vehicle: *n* = 10; scrambled oligo: *n*= 11; JPH4-siRNA2: *n* = 12). *E* and *F*, effect of JPH4 KD on the mechanical (*E*, withdrawal threshold, von Frey method) and thermal (*F*, withdrawal latency, Hargreaves method) sensitivity (vehicle: *n* = 10; scrambled oligo: *n* =12; JPH4-siRNA2: *n* =13). Scatter plots represent data for individual animals; bars represent means; error bars represent SD. [Colour figure can be viewed at wileyonlinelibrary.com]

In the next series of experiments we asked if JPH4 knockdown and impaired SOCE would affect the ability of BK to generate pain *in vivo*. Indeed, BK-induced nociception depends, at least in part, on the ER Ca^2+^ release and subsequent activation of excitatory Ca^2+^-activated Cl^–^ channels (CaCCs) and suppression of inhibitory M-type K^+^ channels (Liu *et al*. 2010; Petho & Reeh, 2012; Lee *et al*. 2014). Thus, we reasoned that since JPH4 knockdown impairs STIM1-Orai1 clustering and inhibits SOCE, it may also affect the ability of BK to sustain inflammatory nociception *in vivo*. To knock-down JPH4 *in vivo* we used JPH4 siRNA2 oligo conjugated to cholesterol for *in vivo* RNA interference (Wolfrum *et al*. 2007) and performed intrathecal injections to the L5/L6 area for DRG delivery (see Methods). Cholesterol-conjugated JPH4 siRNA2 efficiently knocked down JPH4 expression in DRG culture *in vitro* (Fig. 10A). Intrathecal application of siRNA induced marked down-regulation of JPH4 in the whole DRG *in vivo* by 39.5% (*n* = 11; *P*= 0.000293, Kruskal-Wallis ANOVA with Mann-Whitney test; Fig. 10B). Next, we performed hind-paw injections of BK to rats, which received either intrathecal JPH4 siRNA2, scrambled oligo or a vehicle, and analysed pain-related ‘nocifensive’ behaviour (time spent licking, biting and flinching the injected paw). Nocifensive behaviour after the BK injection (10 nmol/site; 50 *μ*l) in either of the control groups lasted for about 15 min. We binned the time after injection in 2-min intervals and calculated the mean time of nocifensive response within each 2-min interval (Fig. 10C). Interestingly, the initial (peak) response to BK was not affected by the JPH4 knockdown, but the effect tailed off significantly faster in JPH4-knocked-down animals, compared to either of the controls (Fig. 10C); as a result, the total time of nocifensive behaviour induced by the BK injection was also significantly reduced (Fig. 10D). This finding is consistent with our hypothesis that JPH4 silencing compromises the formation of SOCE complex in DRG and impairs the ability of DRG neurons to replenish their ER Ca^2+^ stores, thus causing them to desensitise towards the BK-induced excitation much faster. Importantly, general sensitivity of the cutaneous afferents to mechanical or thermal stimulation (as tested by von Frey or Hargreaves tests, respectively; see Methods) was not affected by JPH4 knockdown (Fig. 10E and F). In sum, the experiments presented in Fig. 10 strongly suggest that JPH4 knockdown *in vivo* impairs junctional, GPCR-induced Ca^2+^ signalling in sensory neurons, hence accelerating their desensitisation in an inflammatory environment.

## Discussion

In the present study we investigated Ca^2+^ signalling in ER-PM junctional nanodomains in peripheral somatosensory neurons. We discovered that JPH4 is the major junctophilin expressed in this type of neuron and that JPH4 is necessary for the formation of SOCE complex at ER-PM junctions in response to GPCR-induced ER Ca^2+^ store depletion. Furthermore, we demonstrate a key role of JPH4 and ER Ca^2+^ stores in the maintenance of bradykinin-induced pain *in vivo*. Since the ER supplies Ca^2+^ for the excitatory action of BK and many other inflammatory mediators that are coupled to G_q/11_-types of G*α* protein and PLC-mediated signalling cascades (Linley *et al*. 2010; Liu *et al*. 2010; Petho & Reeh, 2012; Choi & Hwang, 2018), we suggest that junctional Ca^2+^ signalling maintained by JPH4 is an important contributor to the inflammatory pain mechanisms.

Proteins of the mammalian JPH family (JPH1-4) have their N- and C-termini residing in the PM and ER, respectively. The N-terminus contains eight ‘membrane occupation and recognition nexus’ (MORN) repeats, which have high affinity for PM phospholipids and act as a ‘PM anchor’, while the C-terminal trans-membrane segment spans the SR/ER membrane (Kakizawa *et al*. 2007). JPH1 and JPH2 are found in SR-PM junctions of skeletal and cardiac muscle cells, respectively, and facilitate the interaction of PM-localised voltage-gated Ca^2+^ channels and SR-localised ryanodine receptors, thus, orchestrating excitation-contraction coupling (Takeshima *et al*. 2015). JPH3 and JPH4 were mostly found in the CNS but their role is much less understood. It was reported that JPH3/JPH4 double knockout mice exhibited irregular hindlimb reflexes and impaired memory; it was further suggested that these deficits arose from the impaired functional coupling between the NMDA receptors, ryanodine receptors, and SK K^+^ channels due to compromised ER-PM junctions (Moriguchi *et al*. 2006). Another recent study reported that JPH3 and JPH4 are necessary for functional coupling of L-type Ca^2+^ channels, ryanodine receptors and SK K^+^ channels in hippocampal neurons to mediate slow after-hyperpolarisation (Sahu *et al*. 2019). Additionally, JPH4 and junctin were found to form a complex that allows STIM1 recruitment after ER Ca^2+^ discharge in T-cells (Woo *et al*. 2016).

Here we show that JPH4 is abundantly expressed in most (over 75%) DRG neurons, JPH2 is virtually absent whilst JPH1 and JPH3 are present but in a smaller proportion of neurons and their immunoreactivity is also less prominent than that of JPH4. These findings allow us to conclude that JPH4 is the main junctophilin isoform present in DRG. Our data are generally consistent with recent unbiased RNA sequencing data from rat and mouse DRG, which all report JPH4 mRNA as most highly abundant, JPH3 at lower levels and almost no expression of JPH2 (Goswami *et al*. 2014; Thakur *et al*. 2014; Sapio *et al*. 2016; Jager *et al*. 2020). These studies also found almost no JPH1 mRNA present in DRG, while our data (Fig. 1A) suggest moderate JPH1 protein levels. The mismatch between the mRNA levels in DRG found in these studies and the protein levels found in our study requires further investigation. Interestingly, a recent single-cell RNA sequencing study confirmed high levels of JPH4 and 3 in DRG neurons but found neither isoform to be abundant in satellite glia, Schwann cells or DRG-localised macrophages (Jager *et al*. 2020), raising the idea that within the spinal ganglia the JPH-supported membrane structures are important in neurons but not in the other DRG-resident cells.

Airyscan imaging revealed a striking pattern of JPH4 localisation in tubular structures running along the PM in DRG somata and co-localisation of JPH4 with both STIM1 and Orai1 at or near the PM. These observations suggest that JPH4 may indeed represent an important mechanism for ER-PM junction formation and maintenance in sensory neurons. Using proximity ligation and STORM, we show that in DRG neurons JPH4 indeed resides in close (less than 40 nm) proximity of both STIM1 and Orai1. Interestingly, ER Ca^2+^ store depletion in response to BK not only stimulated clustering of STIM1 and Orai1, as is expected form the SOCE complex formation, but also significantly increased clustering of JPH4 with either STIM1 or Orai1 (Fig. 7). Thus, it is reasonable to suggest that JPH4 is an important element of SOCE. In support of this idea, siRNA knockdown of JPH4 abolished BK-induced STIM1-Orai1 clustering (Fig. 8) and impaired functional SOCE (as tested with Ca^2+^ imaging; Fig. 9).

Using JPH4 knockdown as a tool to compromise the ER Ca^2+^ store refill, we show that JPH4 deficiency significantly reduces the ability of the ER to sustain Ca^2+^ transients during repetitive store depletion (Fig. 9G and H). Moreover, intrathecal knockdown of JPH4 in DRG *in vivo* significantly shortened the duration of the BK induced pain without affecting the basal thermal or mechanical sensitivity of cutaneous fibres (Fig. 10). The later notion is an important indication that the JPH4 knockdown specifically affects only these aspects of nociceptive signalling, which depend on junctional Ca^2+^ signalling.

It is important to note that the majority of the data presented here were obtained using DRG neuron cell bodies, but inflammatory excitation of peripheral fibres occurs at the nerve endings and the peripheral sections of the nerves surrounded by the inflamed tissue. Several considerations allow us to hypothesise that the effects observed at the somatic levels are also relevant to the peripheral segments of the somatosensory fibres. (i) BK injections are painful, suggesting presence of functional B_2_R at the cutaneous nociceptive terminals (Liu *et al*. 2010; Lee *et al*. 2014). (ii) BK-induced pain, at least in part, depends on the Ca^2+^-induced activation of ANO1 channels, an effect confined to the ER-PM junctions (Liu *et al*. 2010; Jin *et al*. 2013, 2016; Lee *et al*. 2014). (iii) ER, or ‘axoplasmic reticulum’ is abundantly present in C-fibres; it runs up until the proximal segments of the nociceptive free nerve endings (Kruger *et al*. 2003). Finally, (iv) intrathecal knockdown of JPH4 reduces the duration of BK-induced pain, suggesting the importance of junctional Ca^2+^ signalling at the cutaneous nociceptive terminals for the BK-mediated inflammatory nociception. In addition, it is important to recognise that intracellular signalling events at the somatic level in DRG are not irrelevant. DRG neuron somata are increasingly recognised as important regulators of nociceptive input from the periphery to the CNS, as all the spikes originating at the periphery pass through the DRG on the way to the spinal cord and there is robust filtering at the ganglia, regulated by the somata of the individual nerve fibres (Du *et al*. 2014, 2017). DRGs are densely vascularised (Jimenez-Andrade *et al*. 2008) and not protected by the blood-brain barrier (Hirakawa *et al*. 2004), thus, GPCR-mediated junctional Ca^2+^ signalling is likely to have a strong influence on the control over sensory transmission through the DRG.

In summary, this study has identified JPH4 as a crucial element of nanodomain Ca^2+^ signalling at ER-PM junctions in nociceptive sensory neurons and established the importance of such signalling for inflammatory nociception.

## Supplementary Material

Additional supporting information may be found online in the Supporting Information section at the end of the article.

Supplementary Material

## Figures and Tables

**Table 1:** Primary and secondary antibodies used for immuno-labelling

Antibody	Dilution	Supplier/reference	Cat. No.
Rabbit anti-JPH1	1:200	Biorbyt	orb75099
Rabbit anti-JPH2	1:200	Biorbyt	orb75104
Rabbit anti-JPH3	1:200	Biorbyt	orb75101
Rabbit anti-JPH4	1:200	Biorbyt	orb3020
Rabbit anti-JPH4	1:200	Sigma-Aldrich	PRS4933
Mouse anti-STIMI	1:200	Abcam	ab52458
Rabbit anti-STIM2	1:500	Abcam	ab59342
Mouse anti-Orai1	1:500	Abcam	ab175040
Rabbit anti-Orai1	1:500	Alomone	ACC-062
Rabbit anti-Orai2	1:500	Alomone	ACC-061
Rabbit anti-Orai3	1:500	Alomone	ACC-065
Mouse anti-NF200	1:2000	Sigma-Aldrich	N5389
Mouse anti-DYKDDDDK (FLAG®)	1:1000	Cell Signaling	8146
Guinea-pig anti-TRPV1	1:500	Neuromics	GP14100
Donkey anti-mouse Alexa Fluor 488	1:1000	Invitrogen	A-21202
Donkey anti-mouse Alexa Fluor 555	1:1000	Invitrogen	A-31570
Donkey anti-rabbit Alexa Fluor 488	1:1000	Invitrogen	A-21206
Donkey anti-rabbit Alexa Fluor 555	1:1000	Invitrogen	A-31572
Goat anti-guinea-pig Alexa Fluor 488	1:1000	Invitrogen	A-11073

## Data Availability

All data discussed here are presented in the text and figures.

## Key points

- Rat somatosensory neurons express a junctional protein, junctophilin-4 (JPH4)
- JPH4 is necessary for the formation of store operated Ca^2+^ entry (SOCE) complex at the junctions between plasma membrane and endoplasmic reticulum in these neurons.
- Knockdown of JPH4 impairs endoplasmic reticulum Ca^2+^ store refill and junctional Ca^2+^signalling in sensory neurons.
- *In vivo* knockdown of JPH4 in the dorsal root ganglion (DRG) sensory neurons significantly attenuated experimentally induced inflammatory pain in rats.
- Junctional nanodomain Ca^2+^ signalling maintained by JPH4 is an important contributor to th inflammatory pain mechanisms.
